# Dysregulation of the cGAS-STING Pathway in Monogenic Autoinflammation and Lupus

**DOI:** 10.3389/fimmu.2022.905109

**Published:** 2022-05-27

**Authors:** Holly Wobma, Daniel S. Shin, Janet Chou, Fatma Dedeoğlu

**Affiliations:** Division of Immunology, Boston Children’s Hospital, Boston, MA, United States

**Keywords:** interferon, JAK inhibitor, lupus, autoinflammatory, STING-Associated Vasculopathy of Infancy, Copa Syndrome, Aicardi-Goutières Syndrome, STINGopathy

## Abstract

One of the oldest mechanisms of immune defense against pathogens is through detection of foreign DNA. Since human DNA is compartmentalized into the nucleus, its presence in the cytosol heralds a potential threat. The cGAS-STING pathway is one of the most important cytosolic DNA sensing pathways and leads to interferon signaling, inflammasome activation, autophagy, and cell death. While STING signaling is protective at physiologic levels, chronic activation of this pathway can instead drive autoinflammation and autoimmunity. Here we discuss several monogenic disorders of the STING pathway that highlight its impact on both innate and adaptive immunity in the progressive loss of tolerance. The potential relevance of STING signaling in systemic lupus erythematosus is then discussed with a focus on future avenues for monitoring and targeting this pathway.

## Introduction

One of the most ancient mechanisms of immune defense is the ability to detect and respond to foreign DNA (1, 2). The compartmentalization of our own DNA in the nucleus functions as a built-in mechanism for discriminating DNA that is self (nucleus) versus non-self (cytoplasm). In the last decade, we have gained insight into the specific pathways that mediate cytosolic DNA sensing. From this, the stimulator of interferon genes (STING; other names: MYPS, MITA) pathway has emerged as a key, evolutionarily conserved mechanism dating back 600 million years (2, 3). In a sequence non-specific manner, the enzyme cyclic-GMP-AMP-synthase (cGAS) recognizes double stranded DNA and synthesizes the cyclic dinucleotide (CDN) cyclic-guanosine monophosphate-adenosine monophosphate (cGAMP) from ATP and GTP. cGAMP subsequently binds to STING to trigger downstream innate immune activation.

As we have learned more about the STING pathway, we have also begun to understand the consequences of when this pathway fails. Dysregulated STING signaling characterizes several monogenic autoinflammatory conditions due to gain-of-function in STING or loss-of-function in enzymes that clear small amounts of residual cytosolic DNA. While physiologic STING activity is fundamentally an innate immune sensor, uncontrolled STING signaling affects adaptive immunity – specifically, through promoting the development of autoreactive and dysfunctional B and T cells. As ‘DNA overload’ is also thought to be central to the pathogenesis of systemic lupus erythematosus (SLE), excess nucleic acid signaling may represent a shared origin for the development of autoimmunity. Here, we highlight biologic and pathophysiologic concepts shared amongst disorders of the STING pathway resulting in immune dysregulation and loss of tolerance.

## STING Signaling and Regulation

**Figure 1:**
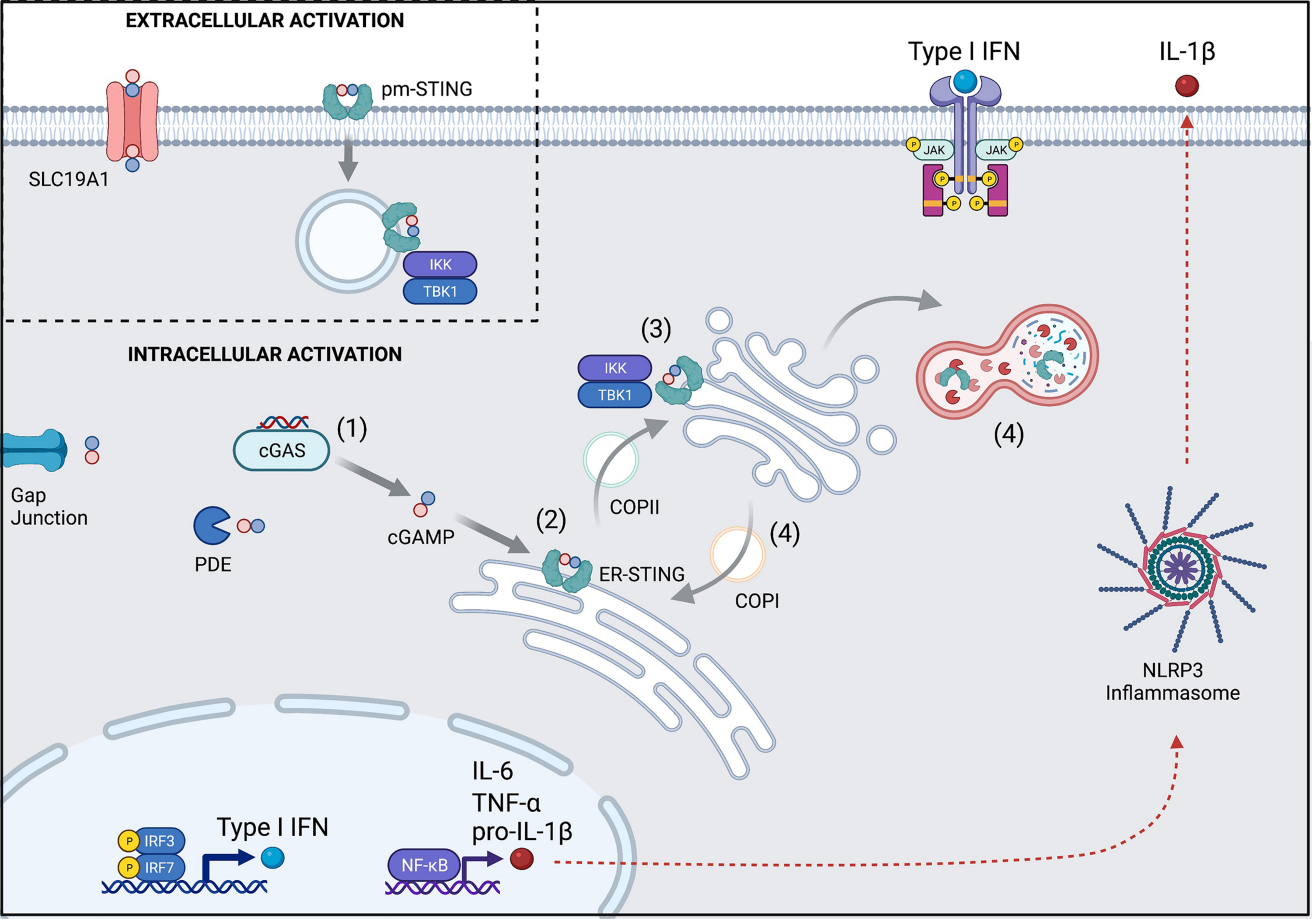
Overview of the cGAS-STING pathway and its regulation. **(1)** cGAS is activated upon binding to dsDNA to synthesize cGAMP from ATP and GTP. Some cGAMP is lost to intercellular spread *via* gap junctions or degradation by phosphodiesterases (PDE), but the remainder can bind to STING. **(2)** cGAMP-bound STING trafficks from the ER to the Golgi apparatus through COPII and becomes activated *en route*. **(3)** Activated STING recruits TBK1 and IKK to upregulate transcription of Type I interferons and NF-κB. **(4)** STING signaling is terminated when it is either degraded in the autolysosome or recycled back to the ER *via* COPI. (Inset) Extracellular cGAMP can trigger STING activation after import by the SLC19A1 transporter or by direct binding to pm-STING. Numbers also refer to stages described in text. pm, plasma membrane; ER, endoplasmic reticulum. Created with BioRender.com.

The classic cGAS-STING pathway can be broken down into four parts (**Figure 1**).

**(1)**First, binding of double stranded DNA (dsDNA) allosterically activates cGAS to make cGAMP. cGAS contains two major DNA binding domains as well as one nucleotidyltransferase (catalytic) domain. It normally exists in an autoinhibited state with inhibitory post-translational modifications, such as phosphorylation at Ser305 and glutamylation (4, 5). When cytosolic DNA is present, such as in the setting of infection, cGAS binds to dsDNA to create a 2:2 complex (6). This enables a conformational change in the catalytic domain resulting in the synthesis of cGAMP from ATP and GTP.
**(2)** Next, cGAMP binds to STING. Human STING is a 379 amino acid (42kDa) protein that is encoded by *transmembrane protein 173* (*TMEM173*). STING is comprised of four transmembrane domains that anchor the protein to the endoplasmic reticulum (ER) where it interacts with the calcium sensor stromal interaction molecule 1 (STIM1). The apo form of STING exists as a homodimer that has a central binding pocket. When cGAMP is present, a single cGAMP molecule binds to this region, inducing a conformational change in the STING homodimer that permits trafficking and downstream signaling.
**(3)** Activated STING initiates an inflammatory response. STING dimers traffic from the ER to the ER-Golgi-Intermediate Complex (ERGIC) through coatomer protein complex II (COPII) vesicles and then to the Golgi apparatus, where glycosaminoglycans enable it to polymerize along the Golgi surface (6). Once at the Golgi apparatus, post-translational modification by palmitoylation at Cys88 and Cys91 facilitate its activation. Activated STING recruits IκB kinase (IKK) and TANK-binding kinase 1 (TBK1). These then activate interferon regulatory factor 3 (IRF3) and Nuclear factor kappa B (NF-κB), which travel to the nucleus to induce type I interferons (IFNα and IFNβ), NOD-, LRR-, and pyrin domain-containing 3 (NLRP3) inflammasome activation, and autophagy. Interferon production contributes to an anti-viral response, while inflammasome activation can trigger inflammation and cell death pathways. Autophagy is thought to help with clearance of cytosolic DNA (negative feedback loop) (4, 5).
**(4)** STING is ultimately degraded by autophagy (7) or recycled back to the ER through coatomer protein complex I (COPI) vesicles, mediated by the UNC-51-like-kinase (ULK1; a serine-threonine kinase) and the lipoprotein cargo receptor surfeit locus protein 4 (SURF4), respectively (4).

Beyond intracellular CDN detection, there have been recent insights towards STING activation by extracellular CDNs (**Figure 1** inset). The membrane channel solute carrier family 19 member 1 (SLC19A1), more commonly known for the ability to import folate, has been shown to also import cGAMP (8, 9). Extracellular CDNs (like cGAMP), once imported, can help to induce a conformational change in cGAS that facilitates its binding to DNA and new cGAMP generation. In this way, extracellular CDNs indirectly contribute to intracellular STING signaling (10). Additionally, alternative splicing of *TMEM173* has recently been described leading to a plasma membrane version of STING (pm-STING) that consists of a single transmembrane domain. pm-STING appears to be internalized after extracellular CDN binding and can similarly lead to recruitment of IKK and TBK1 (11). Extracellular CDN sensing may contribute to detection of bacteria, dying cells, and/or tumors.

Lastly, cGAS and STING may also participate in detection of endogenous nuclear DNA in pathogenic states (5). Uncontrolled DNA breakage events due to genomic instability or genotoxic medications can trigger immediate (within hours) cGAS-independent STING activation due to ubiquitination of STING by TNF receptor associated factor 6 (TRAF6). Following this (within days), the damaged DNA forms micronuclei that can rupture into the cytoplasm, allowing host dsDNA to trigger cGAS-dependent STING signaling (12). These mechanisms serve an important role in anti-tumor immunity. However, in a more insidious manner, higher frequency DNA breakage events with aging may contribute to STING-dependent “inflammaging” and cellular senescence (5).

Activity of the cytosolic cGAS-STING pathway depends on numerous factors. In the healthy state, cGAS levels are negatively regulated by post-translational modification and autophagy. However, upon initial activation, such as in the setting of infection, interferon produced by STING signaling increases cGAS expression as a means of signal amplification. cGAMP levels are controlled by the rate of production by cGAS as well as the rate of degradation and spread. Degradation of cGAMP is mediated *via* the phosphodiesterase (PDE) ectonucleotide pyrophosphatase/phosphodiesterase I (ENPP1), which can be found on the cytoplasmic membrane and the ER surface. Knock-out or blockade of ENPP1 has been shown to significantly prolong the half-life of cGAMP, and certain pathogens (e.g., Group B Streptococcus) have adapted to express PDEs as a means of immune escape (13). Spread of cGAMP between immune cells can occur *via* gap junctions (14). Finally, STING signaling is regulated by ubiquitination, which can promote STING activation or degradation depending on the specific ubiquitin ligase.

Despite the complex machinery of the STING pathway, the activity of STING in patients is currently only monitored at the level of the interferon signaling cascade, either by detection of type I interferons or, more commonly, the upregulation of interferon stimulated genes or proteins (15).

## STING-Associated Vasculopathy of Infancy (SAVI)

SAVI is an autosomal dominant disorder, initially described in 2014, caused by a gain-of-function of STING activity. It is clinically characterized by vasculitis and prominent pulmonary findings usually within the first year of life (16, 17). Patients develop violaceous, scaly lesions in areas of the body with poor circulation (e.g., cheeks, nose, digits) that can ulcerate. On histology, there are mixed features of leukocytoclastic vasculitis and microthrombotic angiopathy (18, 19). In addition to endothelial and immune cells, lung bronchial epithelial cells and type 2 pneumocytes have high expression of STING, and lung disease is severe with both cystic and hemorrhagic features. Additional features such as polyarthritis (20%) may be present. Laboratory studies often show positivity for anti-nuclear antibodies (ANA), anti-neutrophil cytoplasmic antibodies (ANCA), rheumatoid factor, anti-cyclic citrullinated peptide antibodies, and anti-phospholipid antibodies. There is also an increase in interferon stimulated genes.

**Figure 2:**
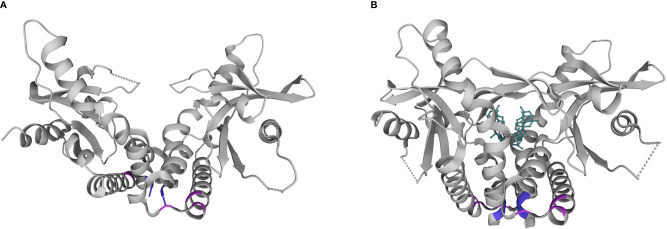
Structural diagram of the STING homodimer in apo **(A)** and substrate **(B)** bound forms. cGAMP is shown in teal. Common locations of pathogenic variants associated with SAVI in the dimerization domain (154, 155; purple) and cytosolic domain (279, 281, 284; pink). Pdb structures 4F9E and 4KSY, respectively. www.wwpdb.org.

The first described SAVI-associated pathogenic variant in *TMEM173* was V155M, thought to stabilize hydrophobic interactions near the dimeric interface in a manner that simulates CDN binding (**Figure 2**). Since then, several additional pathogenic variants have been described that are structurally located in the same region (V147L, N154S). There have also been separate gain-of-function variants described in the cytosolic domain (at positions 206, 279, 281, 284) (19, 20) that may impede inhibitor binding thus stabilizing an activated state (21).

Given that type I interferons are downstream of STING signaling, there has been a historical focus on SAVI as a ‘type I interferonopathy’, supported by the laboratory finding of increased interferon pathway activation in patient samples. For this reason, it was hypothesized that Janus Kinase (JAK) inhibitors could serve as targeted small molecule therapies, given their ability to block interferon signaling. In a recent literature review of SAVI, 10/20 patients who had been treated with JAK inhibitors (ruxolitinib, baricitinib, tofacitinib) showed improvement in their lung disease. Benefit was more consistently seen in patients with variants in the dimerization over the cytosolic domain, although this observation requires further validation with larger patient numbers (21). While type I interferons are upregulated in SAVI, it has become increasingly clear that the pathophysiology of SAVI is very complex, with dysregulation of multiple components of both innate and adaptive immunity. This may explain the inconsistent benefit seen with JAK inhibitors.

In a knock-in mouse model of SAVI (STING N153S) crossed with *Irf3^-/-^*, *Irf7^-/-^*, and *Ifnar1^-/-^* strains, mice proceeded to develop interstitial lung disease and vasculopathy despite a non-functional interferon pathway (22, 23). Meanwhile, if the same experiment were performed using SAVI mice crossed with *Rag1^-/-^* or *Rag2^-/-^* mice lacking T cells, lung disease was abrogated. Since STING is expressed in both hematopoietic and non-hematopoietic lineages, bone marrow chimera experiments were performed to determine if transfer of healthy or mutant marrow was sufficient to rescue or cause disease, respectively. Transplantation of wild-type bone marrow into irradiated STING N153S mice reduced interferon expression but did not protect against lung disease and lethality (22). This was explained by preceding lung damage and residual radioresistant, autoreactive T cells. A separate knock-in mouse model of SAVI using the V154M mutant demonstrated the pathogenic effects of constitutive STING activity within the hematopoietic lineages, as transfer of V154M bone marrow into wild-type mice recapitulated lung disease with donor T cells showing markers of immune activation (24).

Given that mouse models have demonstrated that the lung pathology in SAVI can occur in the absence of interferon signaling and be mediated, in part, by T cell activity, the question remains as to how constitutive STING activity modulates the adaptive immune system. STING is highly expressed in the lung epithelium, such that gain-of-function could lead to lung-specific inflammation. This could include inflammasome activation, as STING has been shown to trigger NLRP3 inflammasome mediated cell death in myeloid cells (25). Gain-of-function in STING appears to promote T cell lymphopenia due to direct effects on lymphoid development in the bone marrow, lymph nodes, and thymus (26, 27) as well as ER-stress induced unfolded protein response and apoptosis (25). However, T cells that are present are more likely to be polarized towards Th1 and Th17 cells, which have been implicated in autoreactivity. Given that cell death often exposes autoantigens, it seems plausible that increased cell death in concert with Th1/Th17 cell polarization could contribute to the development of lung-specific autoimmunity (23).

## Coatomer Protein Complex, Subunit Alpha (COPA) Syndrome

COPA Syndrome is an autosomal dominant disorder initially described in 2015 that was named for a defect in the COPα protein that participates in the COPI heptameric (α,β,β’,δ,ϵ,γ,ζ) complex involved in Golgi-to-ER trafficking (28). While it is incompletely penetrant, 100% of affected patients develop lung disease (cystic, hemorrhagic, interstitial lung disease), with a smaller subset developing arthritis and kidney disease (~86% and 20%, respectively). On laboratory studies, there may be ANA and ANCA positivity, and interferon pathway genes are upregulated. Since patients have normal levels of the COPα protein, the mutation is thought to lead to a non-functional protein that may behave in a dominant negative fashion – disrupting the formation of the heptameric COPI complex. Early reports suggested disease pathogenesis was due to increased ER stress [akin to COPγ deficiency (29)] as well as aberrant autophagy (28). However, due to the similarity of the early onset cystic and hemorrhagic lung disease between COPA syndrome and SAVI, a hypothesis arose that COPA syndrome might also involve the STING pathway (30).

In the last two years, several papers have shown evidence to support this hypothesis. It has been shown that patients with COPA syndrome have cGAS and STING dependent increases in interferon pathway activation and that STING spends a proportionally greater time in the activated state located in the Golgi apparatus (31, 32). This has led to a model of COPA syndrome as ‘the other face of SAVI’ – instead of constitutive ER-to-Golgi forward trafficking *via* COPII in SAVI, there is impaired Golgi-to-ER recycling of STING *via* COPI in COPA syndrome. An important distinction is that disease activity in COPA syndrome may be more sensitive to the amount of cGAMP, as this influences whether there is any activated STING at the Golgi apparatus to become trapped in the first place.

While this interferonopathy model of COPA syndrome neatly connects these two disorders and has led to case reports of using JAK inhibitors (33, 34), as with SAVI, this explanation is not entirely sufficient. A knock-in mouse model of COPA syndrome (E241K) demonstrated that T cells were critical mediators of lung disease, as adoptive transfer of T cells from COPA mice to wild-type mice recapitulated lung disease. The authors further demonstrate that COPA syndrome involves a failure of central tolerance, as mutant COPα leads to impaired negative selection in the thymus resulting in increased development of autoreactive T cells and a corresponding decrease in the regulatory T cell compartment of these clones (35, 36). Concurrent loss of STING activity resulted in the normalization of thymocyte development and T cell aberrations noted in the COPA mutant mice (36). One hypothesis for this failure in tolerance is that impaired autophagy, secondary to dysregulated STING signaling, affects major-histocompatibility-complex mediated antigen presentation in the thymus (35).

Since COPA patients have progressive lung disease even with T cell directed therapy, it seems likely that, as with SAVI, there is an innate driven process within the lung in addition to a generalized loss of tolerance. The lung microbiota in mice has been shown to create a tonic interferon signal, which is thought to confer protection against viruses, but perhaps in COPA patients, the microbiota exacerbates disease activity as it chronically feeds cGAMP into the STING pathway (37). The previously described COPA mouse model was performed strictly in pathogen-free conditions and thus does not fully reflect the significance of bacterial colonization and infection. Assuming that pathogens are an important source of cGAMP in the lung, one could postulate that infection prophylaxis would be an important component to disease management alongside other therapies that target B and T cells, interferon signaling, and autophagy.

## Aicardi-Goutières Syndrome (AGS)

Aicardi-Goutières Syndrome (AGS) is a genetically heterogenous disorder most commonly due to mutations in nucleases and related enzymes (e.g., TREX1, SAMDH1, RNASEH2A, 2B, and 2C) that degrade DNA, RNA, or DNA : RNA hybrids (38, 39). Patients present in infancy with seizures, sterile pyrexia, and developmental regression. While neurologic damage is the most severe and disease defining component of AGS, there may also be vasculitis (particularly chilblains) and later development of autoantibodies and autoimmunity (40).

AGS is associated with increased type I interferon production and was the first disease to bear the classification of ‘type I interferonopathy’ in 2011 (41, 42). Direct, chronic exposure of astrocytes to IFNα causes cellular changes that reduce their stability and proliferation and promote reactive gliosis (43). This may explain why AGS clinically resembles perinatal TORCH infections that also increase the interferon burden from viral infection. Interferon signaling in AGS has been shown to be due to signaling through the cGAS-STING pathway with source DNA likely coming from reverse transcribed retro-elements that are more highly expressed in the brain than other tissues (44, 45). As retro-elements also jump in and out of the genome, they increase the rate of DNA double stranded breaks, which may also increase STING signaling and interferon production. Consistent with this, the level of IFNα in the cerebrospinal fluid is higher in AGS than in SAVI and other type I interferonopathies, whereas the opposite is true for plasma IFNα (46). However, in keeping with other disorders of STING signaling, the disease phenotype of AGS is not exclusively due to interferons, as the presence of T cells is always required in preclinical mouse models.

In a study demonstrating how locally active innate immune activation drives organ specific autoimmunity, investigators developed a mouse model of AGS due to deficiency of the exonuclease Trex1 (47). Unlike in human clinical AGS, which most severely affects the brain, this mouse model generates progressively fatal myocarditis in addition to immune mediated damage to the skeletal muscle, tongue, and skin. *Trex1^-/-^* mice crossed with strains deficient in STING (*Tmem173^-/-^*) or interferon signaling (*Irf3^-/-^*) were rescued from death. This was also true if they were crossed with *Rag2^-/-^* mice, supporting the critical role of T cells in disease pathogenesis. Through a series of bone marrow chimera studies using yellow fluorescent protein (YFP)-Cre reporter mice, the authors demonstrate that the type I interferon expression localized to non-hematopoietic cardiac cells of *Trex1* ^-/-^ mice was sufficient to signal wild-type hematopoietic (antigen presenting) cells to recruit and activate autoreactive T and B cells. These autoreactive cells were then responsible for myocardial inflammation and generation of cardiac-specific autoantibodies. In summary, their model shows that STING-triggered interferon production from non-hematopoietic tissue drives subsequent loss of T and B cell tolerance.

While no AGS model fully captures the clinical phenotype in human disease, the clear interferon signature has led to two trials of JAK inhibitors under an expanded access program (NCT01724580 and NCT03047369) (48). Thirty-five patients were enrolled, initiating baricitinib at a median age of 2.9 years. Twenty patients gained milestones, and twelve patients developed new skills, which was an improvement over their baseline trajectory. In addition to JAK inhibitors, there may be an opportunity for reverse transcriptase inhibitors to prevent generation of cytosolic DNA. Given the rarity of the disease, there are few systematic trials assessing these various therapies (49, 50). It is still debated whether AGS is a progressive or non-progressive disease that occurs after initial neurological insult. Regardless, the window of opportunity for attaining maximal benefit appears to be short, as damage to the brain is thought to be mostly irreversible (50).

## DNAseII Deficiency

DNAseII deficiency was first clinically described in 2017. It results from loss of function of the DNAseII nuclease that degrades dsDNA in the lysosomal compartment. Thus far, four patients from three kindreds have been described with biallelic hypomorphic variants in the *DNASE2* gene (Y95C, G116A, and D121V) (51, 52). Consistent features across patients are neonatal anemia and cholestatic hepatitis. These features resolve in the first few months, with later features variably including autoinflammation, sterile pyrexia, subcortical white matter lesions, learning difficulties, glomerulonephritis, vasculitis, arthropathy, chronic intestinal inflammation, and – in one case – viral triggered hemophagocytic lymphohistiocytosis.

Mimicking the early features in human disease, *DNase2^-/-^* mice, with no enzyme activity, are embryonic lethal due to severe anemia and liver failure. This lethality can be rescued by additional knockout of STING. While DNAseII is an endosomal nuclease, it is speculated that accumulation of dsDNA in the endo-lysosomes ultimately results in their rupture, exposing it to cGAS in the cytoplasm. This early and consistent phenotype across mice and humans is likely secondary to impaired macrophage phagocytosis of red blood cell (RBC) nuclei extruded during erythropoiesis. This leads to DNA overload, inflammation, and cell death in the liver where fetal/neonatal RBCs are produced.

Curiously, *DNase2^-/-^Ifnar1^-/-^* mice, deficient in interferon pathway signaling, also survive but develop an inflammatory arthritis that is based on residual STING-dependent NF-κB pathway activation (53). As arthritis is only one of several later features described in human patients, there are likely both interferon-dependent and independent contributions to the development of systemic autoimmunity. Further studies are required to clearly demonstrate loss of T and B cell tolerance, but it is likely that DNAseII deficiency follows the pattern of our previously described disorders of inflammation unveiling autoreactive lymphocytes.

Given the few reported patients and heterogenous presentation, there is no consistent treatment regimen. The JAK1/2 inhibitor baricitinib was tried in one out of the four described patients and led to clinical improvement in cell counts, gastrointestinal symptoms, and endocrine function.

## Systemic Lupus Erythematosus

Systemic lupus erythematosus is the prototypical multisystem autoimmune disease that is heterogenous in presentation and likely in pathophysiology. Patients may develop autoantibodies, cytopenias, vasculitis, serositis, arthritis, nephritis, rashes, and neuropsychiatric features (54). Lung disease is less common but increasingly recognized, and diffuse alveolar hemorrhage is a rare but potentially fatal complication (55, 56). Unlike in the previously described disorders, there is no single genetic trigger of innate inflammation that leads to organ specific autoimmunity. Despite this, lupus has features of a type I interferonopathy. Aberrant nucleic acid signaling leads to increased interferon production, maturation of plasmacytoid dendritic cells (pDCs), and promotion of autoreactive B and T cells.

There are numerous potential sources of pathogenic nucleic acids in lupus, including chromatin from damaged cells, mitochondria, reverse transcribed RNA, and neutrophil extracellular traps (57). Much focus has been placed on toll-like receptor (TLR)9 as a sensor of endocytosed DNA and DNA-containing immune complexes, specifically in pDCs (58, 59). Given the more recent description of the STING pathway, studies of its relevance in lupus are still in their infancy. However, recent work suggests that it may contribute to disease pathophysiology for some patients with lupus, particularly in sensing mitochondrial DNA that has failed to be cleared by autophagy (60, 61) and dsDNA present in apoptotic derived membrane vesicles (62).

Two different studies look at inhibition of STING signaling in the *Fcgr2b^-/-^* model of lupus, in which deficiency of an inhibitory Fc receptor predisposes to autoimmunity. One study examines this using a STING antagonistic peptide ISD017 and the other study with a knock-out mouse model (63, 64). In both models, the lupus phenotype was ameliorated. All direct effects of STING signaling were blocked, as was downstream pDC maturation and spontaneous B cell germinal center formation (63). Adoptive transfer of dendritic cells exposed to STING ligands into *Fcgr2b^-/-^ STING^gt/gt^* (STING “golden ticket” variant with I199N missense mutation and no detectable STING activity) mice resulted in expanded effector memory T cell populations in the spleen and immune complex deposition in the kidney. This implied that STING-activated DCs were key contributors to loss of tolerance in lupus. To complicate the picture, a different mouse model of lupus showed the opposite effect of STING deficiency on lupus disease activity. In the *MRL.Faslpr* model, in which a mutation in FAS permits survival of auto-reactive lymphocytes, STING (but not IRF3) deficient mice had shorter lifespans, worse lupus nephritis, and expansion of inflammatory myeloid cell populations. Macrophages were hyper-responsive to TLR ligands due to decreases in negative regulators of TLR signaling such as A20 and suppressor of cytokine signaling 1 and 3 (SOCS1, SOCS3) (65). The conclusion drawn from this study was that STING may negatively regulate TLR-dependent immune activation in lupus.

These opposing data reflect the variation and limitation of lupus mouse models, but they are also not entirely surprising. TLR9, which has long been studied as an important nucleic acid sensor in lupus, when entirely deleted in preclinical mouse models (similar MRL genetic background), also led to paradoxical exacerbation of lupus disease activity and decreased survival (57, 66). This could remain consistent with the hypothesis that STING and TLR9 pathways cross-regulate each other such that total deletion of one is still deleterious due to overactivation of the other (66).

Given the limitations of modeling lupus, it is difficult to know how these preclinical models translate to human disease. As both TLR and STING signaling have been implicated in lupus, and they share several downstream effects, a reasonable approach has been to utilize therapies that “cover both pathways” (67, 68). Hydroxychloroquine is thought to impair both TLR and STING signaling through effects on endosomal acidification and cGAS-DNA binding, respectively. It is considered as the cornerstone of lupus therapy upon which immunosuppressive agents are added when needed. An alternate approach is to target the interferon signaling that both TLR and STING pathways converge upon (69, 70). In a phase II trial targeting extrarenal manifestations of lupus, baricitinib showed modest benefit in improving symptoms of arthritis and rash (71). Meanwhile, in the Treatment of Uncontrolled Lupus *via* the Interferon Pathway (TULIP)-2 trial of the IFNαβ receptor antagonist, anifrolumab, there was a greater British Isles Lupus Assessment Group (BILAG)–based Composite Lupus Assessment (BICLA) response rate in the anifrolumab group *vs*. placebo (47.8% *vs*. 31.5%; p=0.001). Response to anifrolumab did not differ by high versus low interferon gene expression at baseline (72). This suggests there is some benefit to direct interferon inhibition, but either interferon gene expression does not predict disease activity, or the anifrolumab dose used in the trial exceeded what was necessary to block interferon signaling in both groups.

Ultimately, these preclinical and clinical studies demonstrate that the role of the TLR9 and STING pathways in lupus pathogenesis is more complicated than two inflammatory cascades converging into a singular interferonopathy. While the pathogenic role of nucleic acids in lupus is generally undisputed, further studies are needed to elucidate the relative contributions of and cross-regulation between the endosomal dsDNA/TLR9 pathway and the cytosolic DNA/STING pathway in individual patients. Some hypothesize that if the predominant nucleic acid sensing pathway for a given patient could be identified, this could lead to more rationale and targeted therapeutics, particularly as cGAS and STING inhibitors are in development (73, 74). As will be discussed, testing this concept is currently challenged by our limited techniques for studying specific nucleic acid sensing pathways in patients.

## Discussion

The cGAS-STING pathway is one of the most ancient defense mechanisms for detecting and eliminating foreign DNA. However, uncontrolled activity in this pathway leads to chronic inflammation and the development of autoimmunity. Here, we have described monogenic defects that affect STING signaling at three different stages. With SAVI, there is gain-of-function in STING leading to uncontrolled STING signaling. In COPA syndrome, there is impaired STING recycling amongst other stressors from impaired Golgi-to-ER trafficking due to the effect of a pathogenic COPA variant on the COPI trafficking complex (75). In AGS and DNAse II deficiency, there is a monogenic defect in nucleic acid clearance leading to excessive nucleic acid input. Similarly, excess nucleic acids in lupus are thought to lead to enhanced cGAS-STING signaling, although this is not an exclusive nucleic acid sensing pathway in this highly heterogenous disease.

**Figure 3:**
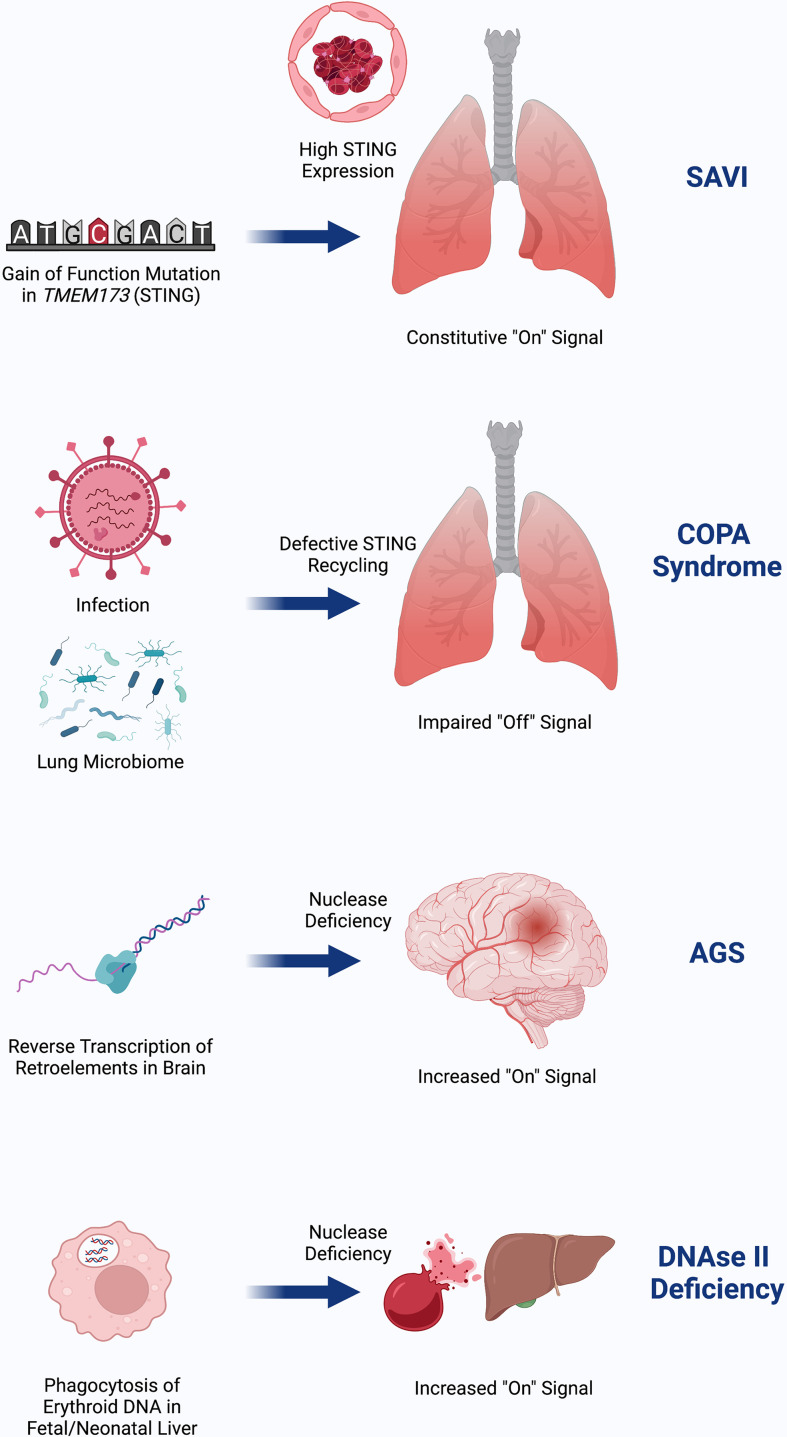
SAVI is due to gain-of-function in STING that is highly expressed in the lung and endothelium, possibly as a means of viral defense. COPA Syndrome disease activity depends on input of DNA, which may come from tonic microbiota signaling in the lung and is worsened in the setting of infection. Failed recycling of activated STING due to pathogenic COPA variants results in excess STING signaling. AGS is due to accumulation of nucleic acids in the brain due to failed degradation of DNA made from reverse transcribed retroelements. This DNA burden then increases STING signaling. DNAseII deficiency initially presents with severe anemia and liver dysfunction due to inflammation from failed ability of macrophages to digest RBC nuclei in the lysosome where DNAseII operates. Similar to AGS, failed clearance of DNA results in increased STING signaling. Created with BioRender.com.

Despite the similar terminal mechanisms of SAVI, COPA, AGS, and DNAseII deficiency, it is not known why these diseases affect different organs (**Figure 3**). The clinical phenotype of SAVI may result, in part, from high expression of STING in the lungs and vasculature (16). COPA syndrome, being more dependent on the presence of basal cGAMP to generate tonic STING signaling, may be influenced by the lung microbiome and infections in the setting of higher baseline STING expression (37). AGS may predominantly affect the brain due to a high burden of RNA retroelements that are reverse transcribed into cytosolic DNA in the brain (38, 39, 45). As with AGS, the presenting clinical phenotype of DNAseII deficiency is sensitive to where the burden of nucleic acids exists, which appears to be in liver macrophages during fetal erythropoiesis. Studies of how STING signaling factors into lupus phenotype are underway.

Given the common feature of these disorders of an increased type I interferon response, they have often been categorized as ‘interferonopathies’. However, this term dates back over 10 years before nucleic sensing pathways like the cGAS-STING pathway were fully described (42, 76). We now know that the STING pathway has pleiotropic effects including those on the inflammasome, autophagy, and cell death. All mouse models described for these diseases are dependent on the role of T cells in disease pathophysiology, thus highlighting the significant contributions of adaptive immunity. Furthermore, as described earlier, there are important interferon-independent effects (77). Rather than ‘interferonopathy’, reframing these diseases as due to aberrant STING-mediated sensing of nucleic acids (we propose ‘STINGopathies’) would more fully encompass the complexity and root cause of these disorders.

This focus on interferon production, which is admittedly built into the name for STING, also reflects the limitations of our own ability to study this pathway in people. Most clinical immunology testing is based on the quantification of proteins rather than unstable molecules like CDNs and cytosolic DNA. Since interferon production is downstream of nucleic acid sensing, an increasingly common assay is to use an ‘interferon signature’ or ‘interferon score’, which quantifies the expression of interferon-stimulated genes (ISG) or proteins (14). This can be done by RNA-seq or flow cytometry. However, upregulation of ISGs is not specific to the STING pathway and captures only one aspect of STING pathway activation (interferon expression). As seen with the TULIP-2 trial, interferon expression may not track proportionally with disease activity or predict targeted treatment response.

**Figure 4:**
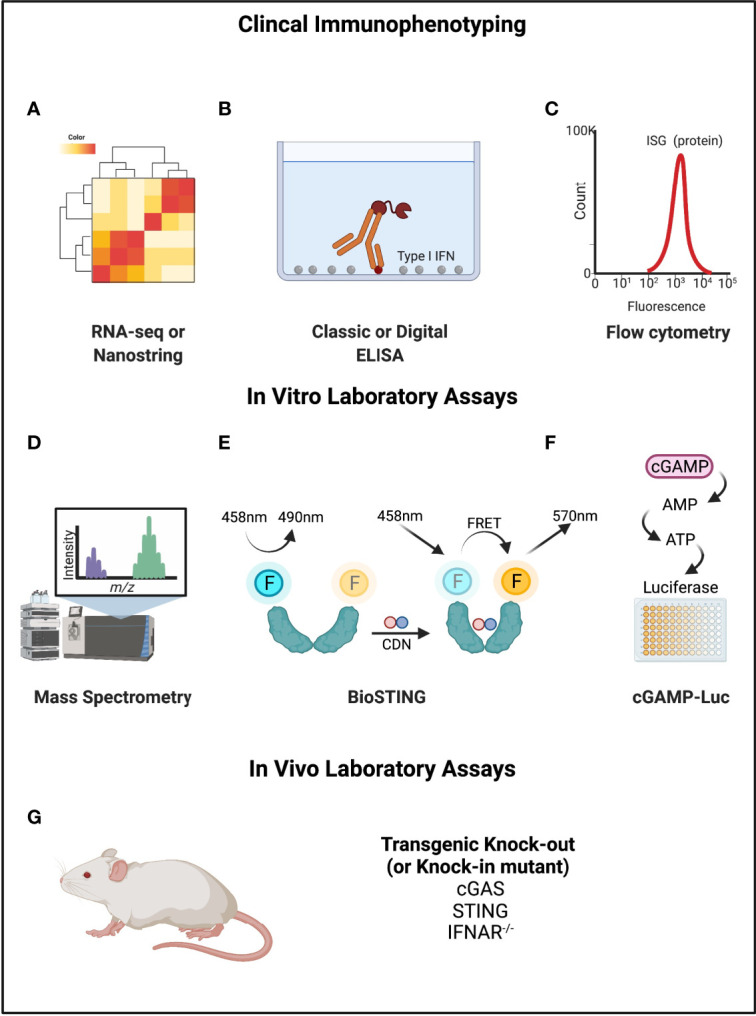
Mechanisms of measuring cGAMP-STING activity. **(A–C)** RNA-seq or Nanostring, ELISA, and flow cytometry can be used to directly detect Type I IFNs or interferon stimulated genes or proteins. **(D)** Mass spectrometry can be used to measure cGAMP. **(E)** BioSTING: when CDNs bind to STING, the conformational change permits FRET such that emission occurs at a different wavelength. **(F)** cGAMP-Luc is based on using ENPP1 to convert cGAMP to AMP (and then ATP), which can be quantified using a standard luciferase assay. **(G)** Mouse models exist for STING pathway knockouts and knock-ins of gain-of-function variants that can be disease causing. Created with BioRender.com.

For this reason, there have been attempts to develop measurement tools that are more specific to STING (**Figure 4**). In a study of cGAS activity in SLE, investigators tried to directly measure cGAMP with ultra-high performance liquid chromatography and mass spectroscopy (78). This identified detectable cGAMP in 15% of SLE patients (7/48) but in no rheumatoid arthritis patients. SLE patients with detectable cGAMP had higher disease activity, but this result was strictly based on the presence or absence, and not the relative amount. Rapid degradation of cGAMP by PDEs was suggested as a reason for the limited signal. Other novel assays include “cGAMP-Luc”, which is a luciferase-based system that depends on the controlled enzymatic conversion of isolated cGAMP to ATP and detection by CellTiter-Glo^®^ (79). Another approach has been named “Bio-STING”, in which STING is engineered to contain fluorophores that act as donors and acceptors of Förster Resonance Energy Transfer (FRET) when the STING homodimer undergoes conformational change from cGAMP binding (80). STING activity can then be detected by fluorescence at the lower energy frequency. Limitations of these newer approaches include their limited availability and practicality for use with patient samples.

There is clearly a great need to have better tools to study nucleic acid signaling. Our initial focus on rare monogenic STINGopathies demonstrates the importance of the STING pathway in the development of organ specific autoinflammation and autoimmunity. For these disorders, we benefit from knowing the specific gene that is disrupted and can generate mouse models with similar defects (albeit imperfect). However, the broader relevance of the STING pathway will come from understanding its role in the context of classic autoimmune disorders. In this review, we focused on lupus as a representative scenario of systemic autoimmunity with a well-established role for aberrant nucleic acid signaling, possibly *via* STING. However, there is emerging evidence that STING signaling may factor into autoimmunity more broadly such as in inflammatory arthritis and dermatomyositis (53, 81–83). Critical outstanding questions regarding its role in autoimmune disease include: In which patients (and diseases) does this pathway play a leading role in disease onset and progression? If we were to develop a clinical test that captures a molecular ‘STING signature’, when would this start to become abnormal? Does this pathway help explain infection-triggered disease flares? Would specifically targeting this pathway ameliorate disease activity? Until we have better tools for assessing STING signaling in patients, it will be hard to answer these questions and rationally apply emerging cGAS-STING targeted therapeutics (73, 74, 84).

## Conclusion

The cGAS-STING pathway is recently recognized as a critical cytosolic DNA sensing pathway. However, uncontrolled signaling leads to increased autophagy, inflammasome activation, and interferon expression with subsequent loss of tolerance to self-antigens. This is best exemplified by monogenic disorders of this pathway, in which patients develop organ specific autoimmunity based on the location of the signaling burden. Future studies and new tools are required to extrapolate these themes to more common, polygenic disorders like systemic lupus erythematosus.

## Author Contributions

HW and DS wrote the draft. JC and FD provided critical edits and feedback. HW and FD conceptualized the paper. All authors contributed to the article and approved the submitted version.

## Conflict of Interest

The authors declare that the research was conducted in the absence of any commercial or financial relationships that could be construed as a potential conflict of interest.

## Publisher’s Note

All claims expressed in this article are solely those of the authors and do not necessarily represent those of their affiliated organizations, or those of the publisher, the editors and the reviewers. Any product that may be evaluated in this article, or claim that may be made by its manufacturer, is not guaranteed or endorsed by the publisher.
